# Juglone Protects Against CLP-Induced Sepsis by Regulating Apoptosis, Pyroptosis, and Oxidative Stress Mechanisms

**DOI:** 10.3390/ph19071130

**Published:** 2026-07-22

**Authors:** Ömer Faruk Başer, Mahmut Karapehlivan

**Affiliations:** Faculty of Medicine, Department of Medical Biochemistry, Kafkas University, Kars 36100, Turkey; mkarapehlivan@gmail.com

**Keywords:** apoptosis, inflammation, oxidative stress, pyroptosis, sepsis

## Abstract

**Background**: Sepsis is a life-threatening systemic condition characterized by organ dysfunction resulting from a dysregulated host response to infection. This study aimed to investigate the protective role of juglone (5-hydroxy-1,4-naphthoquinone), a naturally occurring compound, on lung tissue in a cecal ligation and puncture (CLP)-induced sepsis model. **Methods**: Male Wistar-albino rats were used to establish the model, and juglone was administered intraperitoneally at doses of 1, 2, and 3 mg/kg. Lung and serum samples were collected for biochemical, molecular, and histological analyses through ELISA, RT-PCR, Western blot, and histopathological examinations. **Results**: In the sepsis group, the levels of proinflammatory cytokines (IL-1β, IL-6, IL-18) and pyroptosis-related markers (NLRP3, caspase-1, GSDMD) were significantly elevated, while juglone pretreatment markedly reduced these parameters in a dose-dependent manner. Moreover, juglone upregulated the Nrf2/HO-1 antioxidant pathway while downregulating Keap1 expression. RT-PCR analysis revealed that juglone suppressed the expression of pro-apoptotic genes (caspase-3, caspase-9, Bax) and enhanced anti-apoptotic Bcl-2 expression. Histopathological evaluation demonstrated that juglone alleviated inflammatory cell infiltration, septal thickening, and hemorrhage in lung tissue. **Conclusions**: These findings suggest that juglone is associated with protection against sepsis-induced lung injury and with changes in oxidative stress, inflammation, apoptosis, and pyroptosis pathways. Therefore, juglone may have protective potential against sepsis-induced pulmonary damage.

## 1. Introduction

Sepsis is defined as a highly complex condition in which the host exhibits a dysregulated physiological, biochemical, and pathological response to infection. The organ failure caused by sepsis is associated with high mortality rates. With advancing technology and partial elucidation of the pathophysiology of sepsis, its definitions have evolved. According to the new definition proposed by the Sepsis Consensus, sepsis is described as life-threatening organ dysfunction resulting from a dysregulated host response to infection, while septic shock is defined as a subset of sepsis with circulatory and cellular/metabolic dysfunction associated with a substantially increased risk of mortality [1,2]. Sepsis is the main cause of multiple organ failure, affecting more than 20 million people annually and leading to millions of deaths worldwide. Therefore, the World Health Organization (WHO) has declared sepsis a global health problem and recommended the implementation of coordinated measures to reduce its prevalence and mortality [2,3].

In the pathogenesis of sepsis, the host’s excessive inflammatory response or immunosuppression to infection makes the disease mechanism highly complex. Additionally, oxidative stress and other immunological mechanisms play significant roles in sepsis pathogenesis. It has been reported that factors causing sepsis may lead to organ failure associated with endoplasmic reticulum stress, mitochondrial dysfunction, and tissue damage, accompanied by an excessive inflammatory response mediated by cytokines [4]. Proinflammatory cytokines such as IL-1β, IL-6, IL-8, IL-18, and TNF-α regulate the immune response of the organism and directly influence the prognosis of the disease. These cytokines are stimulated upon encountering pathogenic agents and activate the immune system; however, their excessive expression can amplify the inflammatory response, leading to tissue damage and organ failure, thereby increasing sepsis-related mortality [5].

According to the Surviving Sepsis Campaign 2026 guidelines, the management of sepsis and septic shock encompasses the administration of antibiotics, intravenous fluid resuscitation, corticosteroids, and vasopressors—such as catecholamines—to provide circulatory support. However, these therapeutic modalities present various clinical advantages and disadvantages. The limitations of conventional antimicrobial and hemodynamic supportive agents—including potential toxicity, antimicrobial resistance, ischemia, and fluid overload—coupled with the insufficiency of current adjuvant therapies, underscore a critical scientific and clinical necessity. Consequently, there is an urgent mandate to investigate next-generation therapeutic agents capable of balancing host–pathogen interactions at the cellular level, restoring microcirculation, preventing endothelial damage, and regulating specific immunological and inflammatory pathways [6].

Apoptosis, the fundamental form of programmed cell death, is triggered by extrinsic and intrinsic apoptotic pathways [7]. Experimental animal and human studies have demonstrated that apoptosis is the primary mechanism of cell death in sepsis. Apoptosis also leads to the death of immune cells that fight pathogens responsible for sepsis. The increased survival rates observed in septic animals following inhibition of lymphocyte apoptosis highlight the potential significance of apoptosis in the pathophysiology of sepsis. It has been reported that developing strategies aimed at inhibiting apoptosis could help reduce sepsis-related mortality rates [8].

Pyroptosis is defined as caspase-1-dependent programmed cell death induced by inflammasome activation in various conditions, including bacterial and viral infections, injury, and intoxication. Observed in many different cell types, pyroptosis plays a protective role by triggering the release of inflammatory cytokines and promoting the migration of monocytes to the site of inflammation. Through the targeted destruction of infected cells, pyroptosis facilitates the repair of pathogen-infected cells and prevents the spread of inflammation to surrounding tissues [9].

Oxidative stress is defined as an increase in reactive oxygen species (ROS) production or a decrease in the organism’s ability to eliminate ROS. ROS are generated by various exogenous factors such as airborne particles, chemical exposure, bacteria, and viruses, as well as endogenous factors arising from intracellular metabolic activities [10]. The Keap1-Nrf2-HO-1 signaling pathway plays a key role in the organism’s defense against oxidative stress. Nrf2 is an important transcription factor that suppresses ROS production by regulating the expression of antioxidant enzymes, thereby protecting cells and the organism from inflammation-induced oxidative stress [4].

Juglone (5-hydroxy-1,4-naphthoquinone, 5NQ) is found in the fresh and mature fruit hulls, roots, leaves, and bark of walnut trees. Due to the carbonyl and hydroxyl groups in its structure, juglone exhibits both quinone and phenolic properties. Juglone contains an intramolecular hydrogen bond between its hydroxyl and ketone groups, which it utilizes to scavenge ROS. Various studies have shown that juglone possesses antidepressant [11], dermatological [12], antifungal and antimicrobial [13], antioxidant [14], and anticancer properties [15].

The aim of the present study was to investigate the protective effects of different doses of juglone on lung tissue in a cecal ligation and puncture (CLP)-induced sepsis model through the mechanisms of apoptosis, pyroptosis, oxidative stress, and inflammation.

## 2. Results

### 2.1. ELISA Results

**Figure 1:**
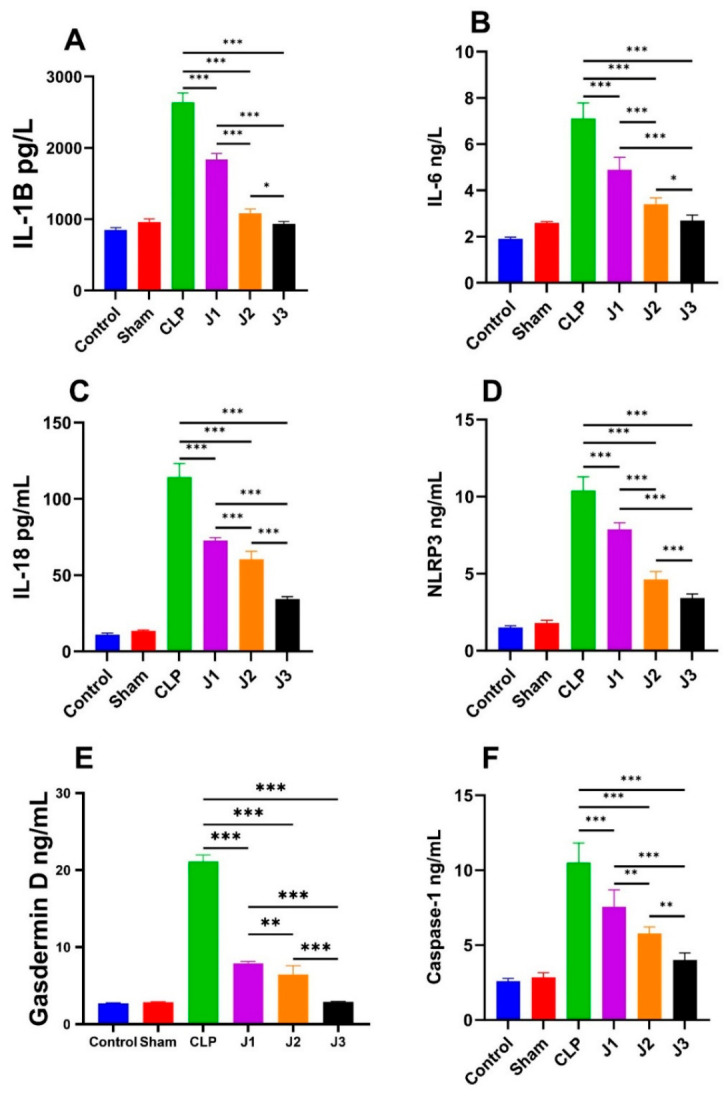
(**A**–**F**) Effects of juglone on serum proinflammatory cytokine and pyroptosis marker levels in CLP-induced sepsis measured by ELISA. Data are presented as mean ± SD. Statistical analysis was performed using one-way ANOVA followed by Tukey’s post hoc test; *p* < 0.05 was considered statistically significant (asterisks denote significant differences vs. the CLP group). * presents *p* < 0.05; ** presents *p* < 0.01 and *** presents *p* < 0.001.

From the serum samples obtained at the end of the study, IL-1β, IL-6, and IL-18 levels were examined to investigate the proinflammatory cytokine mechanism, while the total levels of the pyroptosis-related markers NLRP3, Caspase-1, and Gasdermin-D were measured by ELISA, as shown in Figure 1. Evaluation of the results revealed that the CLP group showed a significant increase in proinflammatory cytokine levels, while these cytokine levels decreased with increasing doses of juglone, used as a protective agent. Furthermore, the serum levels of these pyroptosis-related markers were significantly increased in the CLP group and were markedly reduced with higher concentrations of juglone. The highest levels of NLRP3, Caspase-1, and Gasdermin-D were recorded in the CLP group, whereas these markers decreased significantly with increasing concentrations of juglone. As the ELISA kits used measure total rather than cleaved (active) protein, these data reflect changes in the expression of pyroptosis-related markers rather than direct activation of the pyroptosis pathway.

### 2.2. RT-PCR Results

**Figure 2:**
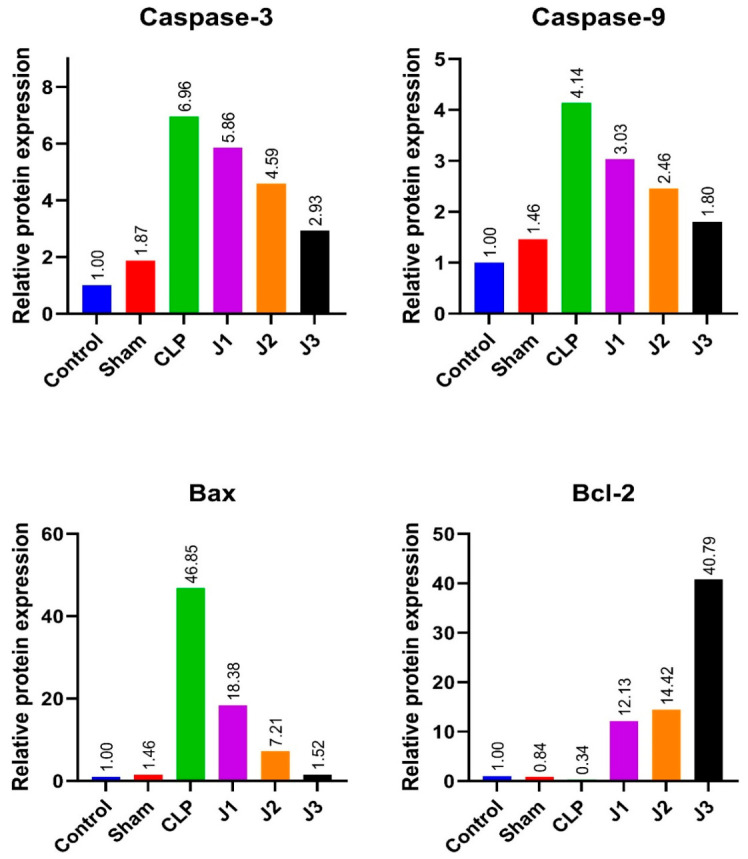
Effects of juglone on apoptosis-related gene expression in lung tissue. mRNA expression levels of the pro-apoptotic genes Caspase-3, Caspase-9, and Bax and the anti-apoptotic gene Bcl-2 were determined by RT-PCR. Results are expressed as fold-change relative to the Control group, calculated using the 2^−ΔΔCt^ method and normalized to reference gene.

RT-PCR analyses were performed on lung tissues collected at the end of the experiment to evaluate the mRNA expression of apoptosis-related genes. Statistical evaluations were made using the control group as a reference and are shown in Figure 2. According to the results, the mRNA expression of the pro-apoptotic genes Caspase-3, Caspase-9, and Bax was highest in the CLP group and decreased with increasing juglone concentration. In contrast, the mRNA expression of the anti-apoptotic Bcl-2 gene was highest in the Juglone 3 group. Of note, Bcl-2 expression was not decreased by CLP relative to the control group; therefore, its increase under juglone treatment may reflect compound-induced transcriptional upregulation rather than reversal of a CLP-induced reduction.

### 2.3. Western Blot Results

**Figure 3:**
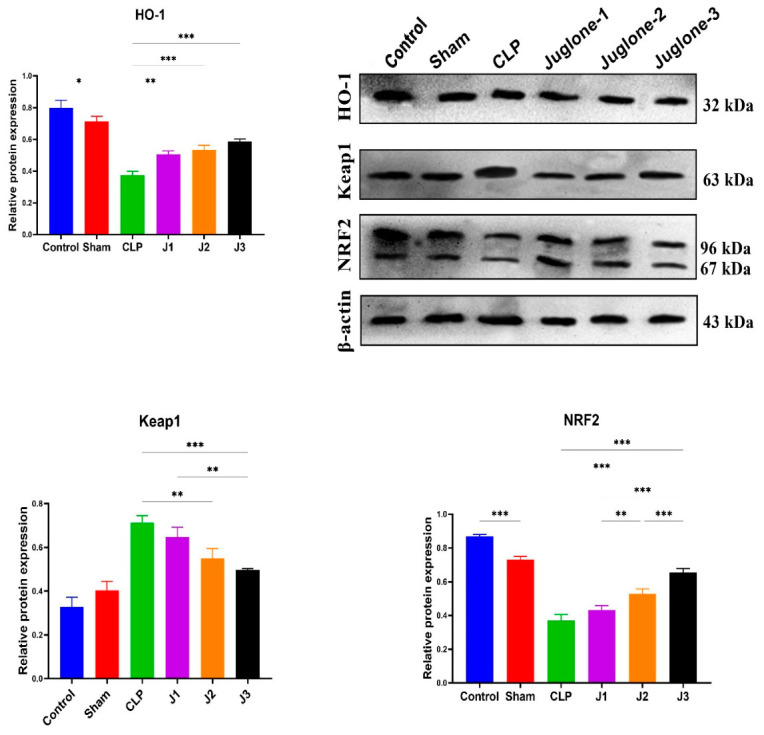
Effects of juglone on Nrf2/HO-1/Keap1 protein expression in lung tissue. Representative Western blot images (upper panel) and densitometric quantification (lower panel) of HO-1, Keap1, and Nrf2. Band intensities were quantified using ImageJ and normalized to β-actin. Data are presented as mean ± SD; one-way ANOVA followed by Tukey’s test, *p* < 0.05 (vs. CLP group). * presents *p* < 0.05; ** presents *p* < 0.01 and *** presents *p* < 0.001.

Western blot analyses were performed on lung tissues collected at the end of the experiment to examine the Nrf2/HO-1/Keap1 antioxidant response pathway. Band images and graphs are shown in Figure 3. According to the obtained results, the group with the lowest HO-1 expression level was determined to be the CLP group, while HO-1 expression was found to increase markedly with higher juglone concentrations. Keap1 expression was highest in the CLP group and was found to significantly decrease in association with increasing juglone concentrations. Similarly, Nrf2 expression was lowest in the CLP group and increased markedly with higher juglone concentrations, paralleling the pattern observed for HO-1. These findings indicate activation of the Nrf2/HO-1 antioxidant response pathway rather than a direct measurement of oxidative stress.

### 2.4. Histopathological and Immunohistochemical Examinations

**Figure 4:**
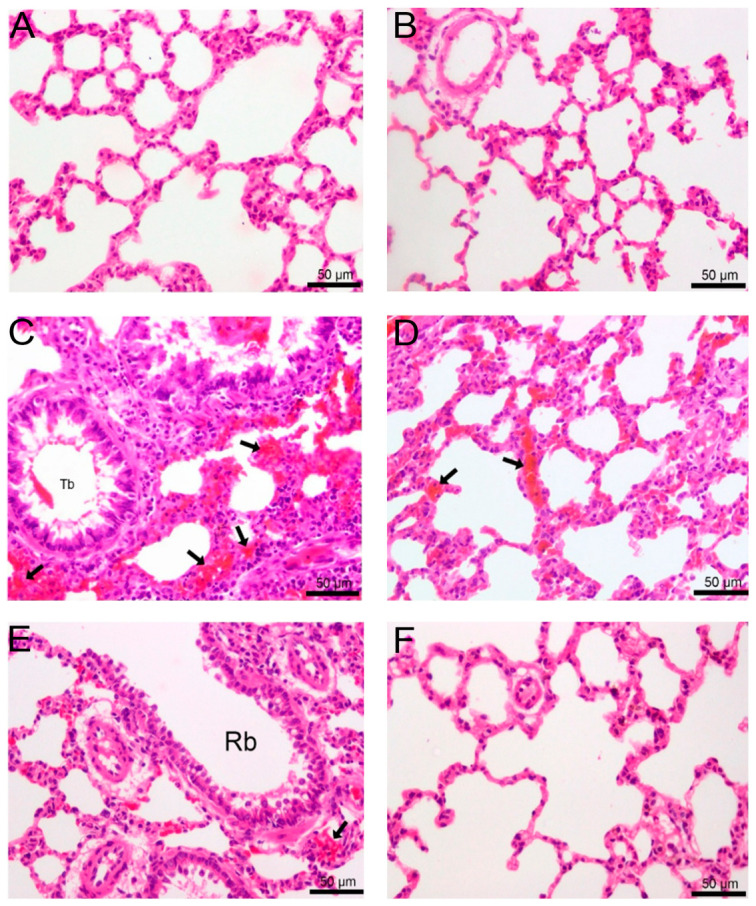
Examination under a conventional light microscope. Staining: Hematoxylin and Eosin. (**A**); Control, (**B**); Sham, (**C**); CLP, (**D**): Juglone 1, (**E**): Juglone 2, (**F**): Juglone 3. Magnification: X40, Abbreviations: Rb: Respiratory bronchiole, Tb: Terminal bronchiole. Black arrow: interalveolar hemorrhage.

For the histopathological evaluation of our study, sections stained with hematoxylin and eosin obtained from all groups were examined in micrographs to assess the presence or absence of interalveolar septal thickening, alveolar contraction, vascular congestion, interalveolar hemorrhage, and areas of inflammatory cell infiltration in lung tissue. Micrographs obtained from the sections of the control group revealed that all lung structures appeared healthy and normal (Figure 4A). Sections from the sham group exhibited a healthy appearance similar to that of the control group, except for some alveolar contractions (Figure 4B). In contrast, in our experimentally induced sepsis model, extensive areas of tissue damage were observed. Thickening of the interalveolar regions, increased inflammatory cell infiltration, vascular congestion, erythrocyte accumulation in alveolar capillaries, and structural deterioration in the epithelium and walls of bronchioles were detected (Figure 4C).

While sections belonging to the Juglone 1 group showed mild normalization compared to the sepsis group, micrographs of lung tissue from the Juglone 2 group were found to be better than those of the Juglone 1 and CLP groups (Figure 4D). Improvement in bronchioles, reduced hemorrhage in interalveolar areas, and decreased congestion in pulmonary vessels were observed (Figure 4E). In the Juglone 3 group, the condition was much better compared to the Juglone 2 group. The damages mentioned above, which were not observed in the control group, were also absent in the Juglone 3 group, and most structures appeared to have a healthy morphology (Figure 4F).

**Figure 5:**
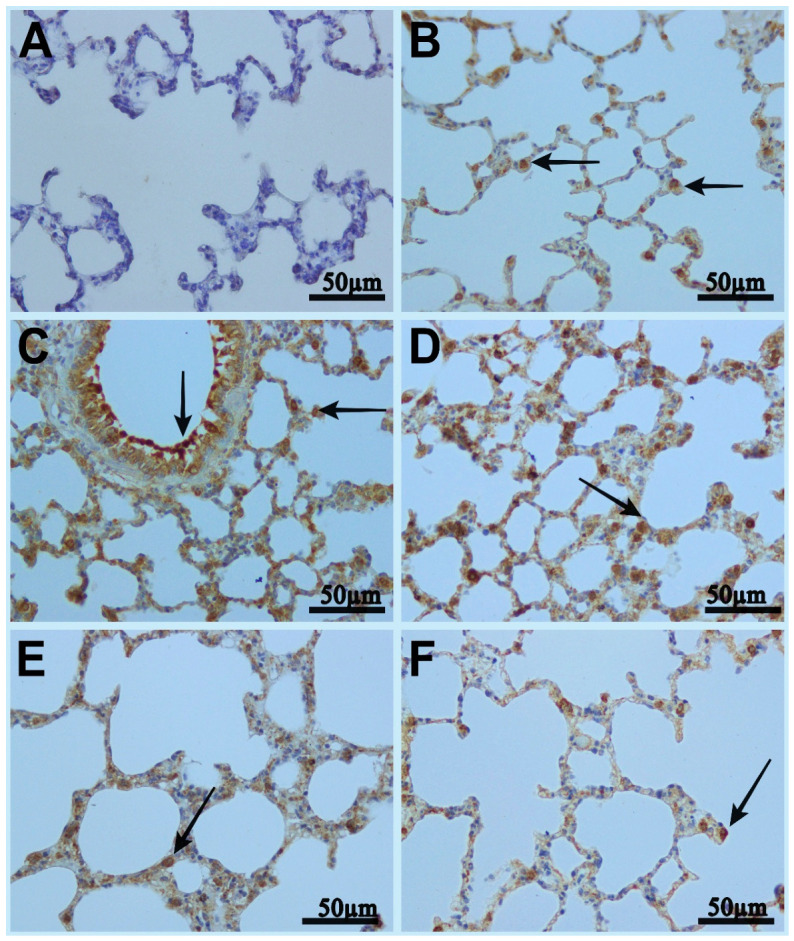
Immunohistochemical examination under a conventional light microscope, Primary antibody: NF-κB. (**A**); Control, (**B**); Sham, (**C**); CLP, (**D**): Juglone 1, (**E**): Juglone 2, (**F**): Juglone 3. Magnification: X10, Black arrow: Immunopositive cells.

In our study, another pathological examination we performed was immunohistochemical labeling using the NF-κB primary antibody, which indicates the cellular reflection of potential damage and the healing process. When we evaluated the results obtained in this context, we found that while the presence of immunopositive cells was very low in the control and Sham groups, there was a marked increase in the CLP group (Figure 5A–C). In the Juglone treatment groups, however, a dose-dependent decrease in the number of positive cells was observed (Figure 5D–F).

All histopathological changes observed in the lung tissue were scored as indicated in Table 1. Histopathological changes were evaluated in a blinded manner and scored semi-quantitatively for five parameters: interalveolar septal thickening, alveolar contraction, vascular congestion, interalveolar hemorrhage, and inflammatory cell infiltration. Each parameter was graded on a 0–3 scale: 0 = absent/normal, 1 = mild, 2 = moderate, and 3 = severe. The scores for the individual parameters were summed to obtain a total histopathological injury score for each animal. ‘−’ presents ‘0’, ‘+’ presents ‘1’, ‘++’ presents ‘2’ and ‘+++‘ presents ‘3’.

## 3. Discussion

Sepsis is defined as a life-threatening abnormal condition that extensively affects physiological and biochemical processes. While the Third International Consensus Definitions for Sepsis (Sepsis-3) defines sepsis as “organ dysfunction caused by a dysregulated host response to infection,” it also emphasizes the role of innate and adaptive immunity in the emergence of clinical symptoms for the first time [1]. Approximately 49 million people are affected by sepsis worldwide annually, resulting in nearly 11 million deaths. It is estimated that sepsis is responsible for approximately 19.7% of global deaths [16]. Despite reports of decreasing mortality rates due to advancing technology and increased access to healthcare, approximately 25% of individuals diagnosed with sepsis still lose their lives. This rate is reported to rise up to 60% in individuals diagnosed with septic shock—severe sepsis accompanied by circulatory and metabolic abnormalities [16,17]. Although the pathophysiological and immunological mechanisms of sepsis have been partially elucidated in recent years, successful therapeutic options specific to sepsis remain limited today. It has been reported that early administration of timely fluid resuscitation and broad-spectrum antibiotics can reduce sepsis-related mortality. In the treatment of sepsis, accurate and early diagnosis, as well as the administration of specific and supportive supplements targeted at the underlying cause, are of paramount importance [18].

The lungs are the first organs affected by sepsis; this condition manifests as acute respiratory distress syndrome (ARDS) and typically results in a mortality rate of around 40% [19,20]. Sepsis causes impairments in lung function by inducing pulmonary exudation through damage to the alveolar walls of the lung tissue. Symptoms of sepsis-induced lung injury include dyspnea, hypoxia, cough, fever, and tachycardia. Furthermore, it is noted that this clinical picture can lead to multiple organ failure, shock, and death, depending on the severity of the sepsis [21,22,23]. Juglone is a compound first isolated from the walnut tree in the 1850s, possessing both quinone and phenolic properties and thought to be beneficial for health [24,25].

Juglone is abundant particularly in the roots, trunks, and leaves of the walnut tree [26]. In vivo and in vitro studies on juglone report antifungal, antimicrobial, anticarcinogenic, antitumor, and antibacterial properties [24,27].

Information regarding the doses used and its toxic threshold in literature reviews is quite complex. In an experimental myocardial injury study conducted in 2022, it was recorded that the most effective dose of juglone in rats was 3 mg/kg via the intraperitoneal (I.P.) route, while 6 mg/kg was noted as toxic [24]. Conversely, in another experimental study, juglone was administered orally at doses of 50, 100, and 150 mg/kg, with 150 mg/kg reported as the most effective dose [28]. In 2024, researchers reported that 10 mg/kg of juglone administered I.P. to mice for 14 days exhibited anticarcinogenic effects [29].

Cytokines are mediators responsible for the control and regulation of immune responses and inflammatory reactions. IL-1β is the first cytokine released in inflammation and induces IL-6 production to facilitate the secretion of acute-phase reactants. IL-1β and IL-6 cytokines increase the severity of inflammation by inducing the release of other pro-inflammatory cytokines. NF-κB functions within the signaling pathway at the nuclear level in the production of these pro-inflammatory cytokines. Additionally, it is known to act as a transcriptional activator that directly affects the inflammatory process and immune response, as well as regulating cell growth and differentiation [30]. While inflammatory cytokines are the most critical mediators of immune system responses, they also govern the initial stage of the inflammatory process. IL-1β and IL-18 are known to be the primary heralds of the immune response [31]. It is reported that IL-1β, IL-18, and IL-6 are inflammatory mediators playing active roles in the autoimmune system and other inflammatory conditions [32,33]. In a study where experimental colitis was induced in 2021, mice were supplemented with 0.04 mg/kg of juglone in their drinking water. Analysis of the data revealed that juglone played an active role in reducing experimental colitis and associated oxidative stress by decreasing serum pro-inflammatory cytokine levels, including IL-1β, IL-6, IL-22, IL-23, and TNF-α [34]. In another experimental study in 2015, rats fed a high-fat diet were orally administered juglone at doses of 0.25 and 1 mg/kg. The results showed a significant increase in key pro-inflammatory cytokines such as TNF-α, IL-1β, and IL-6 in both the serum and hypothalamus of the high-fat diet group, while a remarkable decrease in the expression of these cytokines was observed in the juglone-treated group [35]. In our study, different doses of juglone were administered I.P. to CLP-induced rats; consistent with the aforementioned studies, it was determined that the release of pro-inflammatory cytokines decreased as the juglone concentration increased. The primary mechanism by which juglone reduces pro-inflammatory cytokine secretion is reported to involve increasing the synthesis of antioxidant enzymes via Nrf2 activation, thereby reducing ROS and rendering the organism resistant to pathogens, making immune mechanisms more robust against pathogenic attacks, and inhibiting the NF-κB signaling pathway [29,32,34].

Apoptosis is the fundamental form of programmed cell death and is triggered by extrinsic and intrinsic apoptotic pathways [7]. Experimental animal and human studies have demonstrated that apoptosis is the primary cause of cell death mechanisms in sepsis. Sepsis-induced lymphocyte apoptosis is known to occur via both extrinsic and intrinsic pathways. The increase in survival rates of septic animals upon the prevention of lymphocyte apoptosis highlights the potential importance of apoptosis in sepsis pathophysiology. It has been reported that sepsis-related mortality rates could be reduced through the development of various strategies for apoptosis inhibition [8]. Lymphocytes serve as vital components of the immune system. While the loss of defense system elements such as lymphocytes and macrophages due to increased apoptosis during sepsis reduces the organism’s ability to combat pathogens, it also explains the state of profound immunosuppression in cases of severe sepsis. Furthermore, apoptosis in gastrointestinal epithelial cells can lead to the disruption of intestinal wall integrity, subsequently causing a wider dissemination of pathogens, increasing both the severity of the sepsis and the resulting inflammation. In a 2007 study [36], caspase-3 and caspase-8 genes were silenced using siRNA treatment, followed by the induction of an endotoxic shock model via the CLP method. The study concluded that preventing apoptosis of vascular endothelial cells provided significant protection against polymicrobial endotoxic shock [37]. In a 2024 study, it was reported that juglone treatment in pancreatic cancer cells suppressed proliferation by inducing intrinsic apoptosis through increased ROS-mediated mitochondrial cytochrome-c release [38]. While literature reviews indicate juglone is primarily used in cancer research cell culture models, no studies were found that systematically examine apoptotic mechanisms under sepsis conditions in vivo where systemic integrity is maintained. In this study, juglone administration was associated with reduced mRNA expression of caspase-3, caspase-9, and Bax and increased Bcl-2 mRNA expression. We believe that increased apoptosis leads to a decrease in macrophages, lymphocytes, and dendritic cells, thereby weakening defense and increasing mortality rates due to multiple organ failure. Moreover, we believe increasing concentrations of juglone may activate the Nrf2 pathway, potentially inducing HO-1 and other antioxidant enzymes and thereby enhancing resistance against sepsis pathogens. Our findings, in harmony with literature, support that juglone strengthens immune functions by reducing pro-apoptotic gene expression and supporting cell survival. These results lead us to conclude that juglone plays a protective role in sepsis pathogenesis by regulating host defense. Consequently, we conclude that juglone is associated with protection against sepsis-induced injury, in parallel with reduced pro-apoptotic gene expression. This hypothesis is supported by the work of Bantel et al. [36].

Pyroptosis is defined as caspase-1-dependent programmed cell death, induced by inflammasome activation in various conditions such as injury, toxication, and particularly bacterial and viral pathogens [39,40]. Pyroptosis induces the release of inflammatory cytokines and monocyte migration to sites of inflammation, acting to destroy infected cells to prevent the spread of inflammation [41]. During sepsis, a moderate inflammatory response provides effective resistance against pathogens while ensuring less tissue damage. However, an excessive inflammatory response is noted to lead to secondary infections by suppressing the immune system and allowing pathogens to spread [42]. Pyroptosis has significant functions at different stages of sepsis; while it activates immune cells initially, uncontrolled pyroptosis later worsens prognosis by intensifying inflammatory reactions, leading to shock and organ failure [43]. In a 2020 study, the efficacy of 2.5, 5, and 10 μM applications against LPS and ATP-induced ROS was investigated in the J774.1 cell line. The data showed that while LPS and ATP increased ROS, NO levels, and NLRP3 activation, juglone applications suppressed pyroptosis by inhibiting NLRP3 activation and active caspase-1 formation. Additionally, juglone suppressed the release of pro-inflammatory cytokines such as IL-1β and IL-18, suggesting therapeutic potential in inflammation-related diseases [32]. In an in vivo experimental setup in 2025, an autoimmune prostate model was induced in mice to examine pyroptosis and oxidative stress. Mice were treated with 0.25 and 1 mg/kg juglone via oral gavage. High-dose juglone treatment alleviated prostate infection and pelvic pain by inhibiting NLRP3 and GSDMD-mediated pyroptosis. The same study noted that higher doses decreased IL-1β, IL-6, and IL-18 secretion. It is suggested that juglone reduced pyroptosis and inflammation by suppressing caspase-1 activity, decreasing MDA, and increasing SOD and GPx activity. In vitro results in the same study were similar to in vivo findings [44]. Examination of our study’s data revealed that the administered juglone doses significantly decreased NLRP3, caspase-1, and GSDMD levels compared to the CLP group. This decrease was inversely proportional to juglone concentrations. We believe the excessive increase in these markers in the CLP group causes an exaggerated inflammatory response, weakening the organism and reducing survival. We suggest juglone’s inhibitory effect on NLRP3, caspase-1, and GSDMD may occur via NF-κB regulation and Nrf2 induction, with increased HO-1 and other antioxidant enzymes. Furthermore, we suggest that juglone may help reduce sepsis-induced ROS, thereby enhancing the ability to combat pathogens.

The Keap1-Nrf2-HO-1 signaling pathway plays a crucial role in combating oxidative stress [45,46]. Nrf2 is a vital transcription factor protecting against inflammation-derived oxidative stress [47,48]. LPS suppresses glutathione synthesis—active in antioxidant defense—by inhibiting Nrf2 and MafG protein ubiquitination. This suppression renders cells and the organism more sensitive and vulnerable to ROS. It is noted that inducing Nrf2 activation or removing its inhibition via enzymes like ubiquitin ligase 9 makes the organism stronger against oxidative stress [49]. In the CLP sepsis model, antioxidants like resveratrol, mangiferin, myricetin, and ascorbic acid increase survival by raising Nrf2 expression and reducing tissue damage [50,51,52,53]. Nrf2 activation also exerts a protective effect by enhancing macrophage antibacterial capacity [53,54]. Consequently, the Keap1/Nrf2/HO-1 pathway plays an important role in alleviating sepsis-induced inflammation and damage. Inducing Nrf2 activation carries the potential to improve sepsis prognosis [49]. 0.098Given the close crosstalk between the Nrf2/HO-1 antioxidant response, NF-κB-driven inflammation, and apoptotic and pyroptotic signaling, the changes observed here most likely reflect an integrated response rather than independent effects on each pathway.

This approach has some limitations. Firstly, lower dose trials of juglone could not be studied; activity at lower doses could be evaluated in later studies. Secondly, we only examined lung tissue in this study; examinations in other tissues (spleen, kidney, and liver) could be performed. Thirdly, the pyroptosis-related markers (NLRP3, caspase-1, and GSDMD) were quantified only as total protein by ELISA, and the cleaved/active forms of caspase-1 (p20) and gasdermin D (GSDMD-N) were not assessed. Therefore, our findings indicate changes in the expression of these markers rather than confirmed activation of the pyroptosis pathway. Similarly, apoptosis was assessed only at the mRNA level (Caspase-3, Caspase-9, Bax, and Bcl-2), and the cleaved/active forms of caspase-3 and caspase-9 or their enzymatic activities were not measured. Accordingly, the proposed anti-apoptotic effect is based on changes in gene expression rather than on direct evidence of apoptosis. Future studies using western blotting of the cleaved forms (caspase-1 p20 and GSDMD-N) or caspase activity assays are warranted to directly confirm the proposed pyroptosis and apoptosis mechanisms. Furthermore, oxidative status was evaluated only through the antioxidant response proteins Nrf2, HO-1, and Keap1, whereas direct markers of oxidative stress (e.g., ROS, MDA, SOD, CAT, and GPx) were not measured. Finally, although juglone pretreatment was associated with parallel changes in oxidative stress–, inflammation-, apoptosis-, and pyroptosis-related markers, these processes are closely interconnected, and the present study cannot determine which of them represents the primary molecular target of juglone and which are secondary or downstream consequences of its protective effect. Dedicated mechanistic studies, for example using pathway-specific inhibitors or gene silencing, are needed to define the primary target and the causal sequence of these events.

## 4. Materials and Methods

### 4.1. Ethical Approval of the Study

The ethical approval for this study was obtained from the Kafkas University Local Ethics Committee for Experimental Animals (KAÜ-HADYEK) under protocol number KAÜ-HADYEK/2025-055. The subjects were obtained from the Atatürk University Medical Experimental Practice and Research Center. A total of 44 male Wistar-albino rats, 12 weeks old and weighing 280–300 g, were used in the study. Throughout the experimental procedure, all subjects were housed in rooms maintained at 22 ± 2 °C with a 12 h light/12 h dark cycle and provided ad libitum access to standard rat feed and drinking water. The animals were randomly grouped as follows.

### 4.2. Experimental Design

Animals were randomly distributed into the following groups: Control (*n* = 6), Sham (*n* = 6), CLP (*n* = 8), Juglone 1 mg/kg (*n* = 8), Juglone 2 mg/kg (*n* = 8), and Juglone 3 mg/kg (*n* = 8). Juglone was dissolved in a 1% DMSO solution. Based on the “reduce” principle, one of the 3R rules applied to experimental animals, DMSO was administered to all groups, including the control, to reduce the number of groups and subjects, and the entire experimental setup was standardized. Therefore, there is no separate DMSO group in the study. Prior to the CLP procedure, freshly prepared juglone solution was administered intraperitoneally for seven consecutive days at doses appropriate to body weight, with injection sites rotated. Eighteen hours after the final juglone administration, polymicrobial sepsis was experimentally induced using the cecal ligation and puncture (CLP) method, based on the methodology described in the literature [55]. As a summary, all surgical procedures were performed under aseptic conditions. Rats were anesthetized with intraperitoneal ketamine hydrochloride (100 mg/kg) and xylazine (15 mg/kg). Following the administration of anesthesia, the abdominal area of each rat was carefully shaved, and a midline incision was performed under sterile conditions to access the abdominal cavity. The cecum was subsequently identified, isolated, and ligated using a 3.0 silk suture just distal to the ileocecal valve. Two punctures were made twice on the opposite side of the mesentery with a 16-gauge needle through the cecum distal to the point of ligation, and the cecum was returned to the peritoneal cavity. The abdominal incision was then closed with a 4/0 sterile synthetic absorbable suture in two layers. Immediately after surgery, all animals received warm sterile saline (s.c.) for fluid resuscitation and post-operative analgesia with (buprenorphine, 0.05 mg/kg, s.c.) every 12 h. Animals were monitored for clinical signs, body weight, and sepsis severity according to predefined humane-endpoint criteria. These criteria were based on a clinical severity assessment of appearance, spontaneous activity and posture, response to external stimulation, respiratory rate and pattern, and surface body temperature, together with body-weight change; an animal showing inability to ambulate, agonal or severely labored respiration, marked hypothermia, or body-weight loss greater than 20% was to be euthanized immediately under deep ketamine/xylazine anesthesia. No animal reached the humane-endpoint criteria or died before the scheduled 18 h endpoint. Accordingly, all 44 animals survived to the scheduled endpoint and were included in all biochemical, molecular, and histopathological analyses; no animals or samples were excluded.

Eighteen hours after the CLP procedure, intracardiac blood samples were collected under ketamine/xylazine anesthesia. Blood samples were centrifuged at 3500 rpm for 10 min, and the sera were separated and stored. Portions of the lung tissues obtained from the animals were fixed in 10% formalin for histopathological analysis, while the remaining parts were stored at −80 °C until the day of Western Blot and RT-PCR analyses.

### 4.3. Drugs

Juglone was obtained from BLDPharm (BD12556). DMSO (BioShop, catalog number DMS555) was used to dissolve juglone.

### 4.4. ELISA Analyses

Following the procedures included in the ELISA kits, measurements were performed using a spectrophotometer. Commercial ELISA kits were obtained from YL-Biont. The following parameters were analyzed: Caspase-1 (YLA1380RA), NLRP3 (YLA1805RA), GSDMD (YLA1790RA), IL-18 (YLA-0033RA), IL-6 (YLA-0031RA), and IL-1β (YLA-0030RA). All pipetting procedures were performed using the Automatic Pipetting Robot (PRCXI-SC9300-V03) located in the Molecular Immunology and Stem Cell Laboratory of Kafkas University Faculty of Medicine.

### 4.5. RT-PCR Analyses

At the end of the experiment, the mRNA expression levels of Caspase-3, Caspase-9, Bax, and Bcl-2 genes were analyzed by RT-PCR using lung tissues [17]. RNA isolation was performed using the Allsheng Auto-Pure Automatic DNA/RNA Isolation Robot (Allsheng Instruments Co., Hangzhou, China) with the MagBar 96 RNA Isolation Kit. Cold blocks were used during all stages of RNA isolation. The conversion of RNA obtained from lung tissues into complementary DNA (cDNA) was carried out on an Applied Biosystems™ MiniAmp™ Thermal Cycler (ThermoFisher Scientific, Waltham, MA, USA). The synthesis of cDNA was performed using the 5× First Strand cDNA Synthesis Kit (EcoTech, Erzurum, Türkiye) in accordance with the manufacturer’s protocol. All steps were conducted on cooling blocks.

The mRNA expression levels of the parameters were determined from the obtained cDNA samples using the Real-Time PCR Technique with LongGene Real-Time PCR Systems (LongGene Scientific Instruments Co., Hangzhou, China). EcoTech 2× SYBR Master Mix (EcoTech, Erzurum, Türkiye) was used for real-time PCR analysis. All procedural steps were carried out on a cooling block. After completion of the analyses, the fold-change values were calculated using the 2^ΔΔCt^ method and presented graphically. The base sequences of the primers used in the real-time PCR analyses are provided in Table 2.

### 4.6. Western Blot Analyses

Lung tissue samples were weighed and then ground using liquid nitrogen. Before WB analysis, total protein levels in tissue samples were measured, and a minimum of 40 µg of sample per well was used. A 40 µg protein sample was electrophoresed using 8–12% SDS-PAGE, and the separated proteins were transferred onto a PVDF membrane. Primary antibodies purchased from Santa Cruz Biotechnology (Dallas, TX, USA) were used overnight at 4 °C: Nrf2 (sc-81342), Keap1 (sc-515432), HO-1 (sc-10789), and β-actin (sc-47778). These were prepared in blocking buffer at a concentration of 5 µg/mL, added to the membrane, and incubated overnight in a refrigerator on an orbital shaker. Subsequently, secondary antibodies (sc-2004/sc-2005) prepared in blocking buffer at a concentration of 1 µg/mL were added and incubated at room temperature for 1 h. Finally, the chemiluminescent HRP substrate prepared according to the manufacturer’s instructions was applied to the membrane, and the blots were visualized using a gel imaging system (Bio-Rad, Hercules, CA, USA) in chemiluminescence mode. Normalization was performed by dividing the total protein amount obtained from the gel analysis of a sample by the blot intensity on the membrane [56].

### 4.7. Histopathological Examinations

Fixed lung samples were processed using the paraffin section preparation technique, which includes dehydration, clearing, and embedding in paraffin wax to create block designs. Sections with an average thickness of 5 μm were taken from the paraffin blocks and stained using the following histological staining methods.

Hematoxylin–Eosin Staining: Lung tissue samples were taken and fixed in a 10% formaldehyde solution. The method was applied as described in our previous study. İHC Staining: For analysis, lung tissue was stained for NF-κB (NF-κB, Santa Cruz Biotechnology, sc-109), which initiates inflammation. The method was applied as described in our previous study [57]. Six sections were taken from each subject. Five randomly selected areas from each section were then analyzed by two blind histologists.

### 4.8. Statistical Analyses

GraphPad Prism 10.1 software was used for the statistical analysis of data obtained from our study. To determine whether the ELISA data were normally distributed, the Normality and Lognormality Test (Shapiro–Wilk Test) was applied. Since the data showed a homogeneous distribution, one-way ANOVA analysis followed by Tukey’s test was used (* *p* < 0.05 was considered statistically significant). In Western blot analyses, the band intensities were determined using the ImageJ software (version 1.54p) and normalized to the internal control β-actin (1:1000). RT-PCR analysis results were expressed as fold-change graphs calculated using the 2^ΔΔCt^ method.

## 5. Conclusions

Juglone pretreatment was associated with changes in apoptosis, pyroptosis, oxidative stress, and inflammation pathways, which may enhance the body’s capacity to combat sepsis-causing pathogens and the reactive oxygen species it encounters. In doing so, it alleviates the burden that sepsis imposes on the immune system and supports survival. Based on the obtained data, we conclude that juglone may serve as a novel and natural protective agent against various inflammatory diseases, particularly sepsis.

## Figures and Tables

**Table 1:** Semi-quantitative histopathological scoring of experimental groups.

Examination	Control	Sham	CLP	J1	J2	J3
Interalveolar septal thickening	−	−	+++	++	++	+
Leukocyte infiltration	−	+	+++	++	++	+
Pulmonary edema	−	−	+++	+	−	−
Perivascular edema	−	−	+++	++	++	−
Alveolar macrophage accumulation	+	+	+++	++	++	+
Hemorrhage area	−	−	+++	++	++	+
NF-κB positivity	−	+	+++	+++	++	+

**Table 2:** Forward and reverse sequences of primers used in Real-Time PCR analyses.

*Caspase-3*	Forward Reverse	5′-GTGGAACTGACGATGATATGGC-3′ 5′-CGCAAAGTGACTGGATGAACC-3′
*Caspase-9*	Forward Reverse	5′-AGCCAGATGCTGTCCCATAC-3′ 5′-CAGGAACCGCTCTTCTTGTC-3′
*Bax*	Forward Reverse	5′-CGGCGAATTGGAGATGAACTGG-3′ 5′-CTAGCAAAGTAGAAGAGGGCAACC-3′
*Bcl-2*	Forward Reverse	5′-TGTGGATGACTGACTACCTGAACC-3′ 5′-CAGCCAGGAGAAATCAAACAGAGG-3′
*β-actin*	Forward Reverse	5′-AAGATCCTGACCGAGCGTGG-3′ 5′-CAGCACTGTGTTGGCATAGAGG-3′

## Data Availability

The raw data supporting the conclusions of this article will be made available by the authors on request.
